# Wound Healing Potential of Intermittent Negative Pressure under Limited Access Dressing in Burn Patients: Biochemical and Histopathological Study

**Published:** 2018-01

**Authors:** Honnegowda Thittamaranahalli Muguregowda, Pramod Kumar, Padmanabha Udupa Echalasara Govindarama

**Affiliations:** 1 Department of Plastic Surgery and Burns, Kasturba Medical College, Manipal, Karnataka, India; 2 Department of Plastic Surgery and Burns, King Abdul Aziz Specialist Hospital, Sakaka, Al-jouf Saudi Arabia; 3 Department of Biochemistry, Kasturba Medical College, Manipal, Karnataka, India

**Keywords:** Burn; Wound, ROS, Limited access dressing, Malondialdehyde, Antioxidant

## Abstract

**BACKGROUND:**

Malondialdehyde (MDA) is an oxidant that causes damage to membranes, DNA, proteins, and lipids at the cellular level. Antioxidants minimize the effects of oxidants and thus help in formation of healthy granulation tissues with higher level of hydroxyproline and total protein. This study compared the effect of limited access dressing (LAD) with conventional closed dressing biochemically and histopathologically.

**METHODS:**

Seventy-two 12-65 years old burn patients with mean wound size of 14 cm^2^ were divided to two groups of LAD (n=37), and conventional dressing groups (n=35). Various biochemical parameters were measured in granulation tissue. Histopathological analysis of the granulation tissue was studied too.

**RESULTS:**

LAD group showed significant increase in hydroxyproline, total protein, GSH, and GPx and decrease in MDA levels compared to conventional dressing group. A significant negative correlation between GSH and MDA was noted in LAD group, but in conventional dressing group there was no significant correlation. A significant negative correlation between GPx and MDA was noticed in LAD group, but in conventional dressing group was not significant. There was a histologically fewer inflammatory cells, increased and well organized extracellular matrix deposit, more angiogenesis in LAD group after 10 days while the difference was significant between the groups.

**CONCLUSION:**

Our study showed a significant reduction in oxidative stress biomarker of MDA, increase in hydroxyproline, total protein, antioxidants and amount of ECM deposition, number of blood vessels and a decrease in the amount of inflammatory cells and necrotic tissues in LAD group indicating the better healing effect of burn wounds.

## INTRODUCTION

Burns are a common traumatic injury that results both in local tissue damage and in a systemic mediator-induced response; there is evidence of both local and systemic oxidant changes manifested by increased free radical activity and lipid peroxidation. At the same time burn injury causes a remarkable decrease in total antioxidant status and a reduction in antioxidant scavenging capacity when compared with control.^1^ Auto oxidation processes arise from reactive oxygen species such as hydrogen peroxide, hydroxyl radicals, and superoxide radicals, generated as byproducts of aerobic metabolism or other processes such as thermal injury.^2^

These reactive oxygen species (ROS) cause auto oxidation of polyunsaturated fatty acids leading to the formation of lipid peroxides. Such peroxides have numerous deleterious effects on biological systems^3^ and have been implicated pathogenesis of several diseases.^4^ There is strong evidence that the toxic products of burn oxidative stress are systemically circulating lipid peroxides. Lipid peroxides ultimately give rise to MDA that has been shown to react with proteins^5^ and amino acids.^6^

One study reported a significant increase in lipid peroxide levels, as measured by MDA concentration, in the serum of rats and mice, 4 h after burn injury to the skin.^7^ An increased level of MDA was found in the plasma of burn patients shortly following burn injury, and lasting up to 12 days post injury.^8^ Among the major defense processes which combat the deleterious oxidation effects resulting from reactive oxygen species are reduced glutathione (GSH), certain glutathione-dependent enzymes; such as glutathione peroxidase (GPx), glutathione -S- transferase, and glutathione regenerative enzyme, glutathione reductase.^9^

GPx, a selenium –enzyme, catalyzes the reduction of hydrogen peroxide, organic peroxides,^10^ GPx, along glutathione reductase, serves to detoxify a major portion of the cellular hydro-peroxides and peroxides generated by reactive oxygen species.^11^ Certain forms of glutathione -S- transferase, in addition to catalyzing conjugations of glutathione with toxic compounds (xenobiotics), including the degradation product of arachidonic acid, 4-hydroxynon-2-enal^12^ have also been shown to catalyze the reduction of organic peroxides.^13^ Depletion of cellular reduced glutathione (GSH) may reduce the cellular ability to destroy free radicals and reactive active species, thereby raising the general oxidative potential in the cells. Oxidized glutathione (GSSG) is a physiological indicator of the activity of the intracellular defense system against reactive oxygen, and it can be used to monitor oxidant stress in vivo.^14^

The biological processes for repair of burn wounds are unlikely to be different from those of any other wound,^15^ many strategies have been developed to try to manipulate this wound healing process,^16^ and to minimize the progression of burn wounds by involving deeper tissue in the acute phase. These strategies range from use of a variety of skin substitutes and dressings, such as polyurethane films and hydrocolloids,^17^ to the use of more complex and experimental techniques, such as hyperbaric oxygen therapy,^18^ application of growth factors and cytokine biology.^19^ One way of removal of harmful chemicals along with wound exudates and manipulating the wound environment with a view of promoting healing is to apply negative-pressure wound therapy (NPWT) across the wound surface via a dressing.^20^

Burn treatments include a variety of dressings, as well as newer strategies, such as NPWT, which, by means of a suction force that drains excess fluids from the burn, tries to promote the wound healing process and minimize progression of the burn wound. Negative pressure is purported to induce an interstitial gradient shift which can cause a reduction in oedema, and a secondary increase in dermal perfusion, thus aiding in the removal of blood or serous fluid.^21^ It is also postulated that the ability of NPWT to produce a mechanical stress or force that has a direct effect in cellular activity due to increased levels of growth factors and development of new blood vessels and this contribute to a decrease in burn wound progression.^22^ The maintenance of a moist environment that provides optimal conditions for epithelialization and the prevention of tissue desiccation.^23^

 Limited access dressing (LAD) is a combination of intermittent NPWT and moist wound healing (cycle of 30 minutes suction and 3^1^/_2_ hours rest).^20^ The present study evaluates role of LAD on 10-30% total body surface area burn wounds of more than 6 weeks duration. Various parameters studied were levels of hydroxyproline, total protein, malondialdhyde, reduced glutathione, glutathione peroxidase.

## MATERIALS AND METHODS

The study is prospective randomized clinical trial which was carried out in the Department of Plastic Surgery and Burns, Kasturba Hospital, Manipal, India. Institutional Ethics Committee of Kasturba Medical College and Hospital, Manipal University approved the study protocol and study was registered to Clinical Trials Registry India, (Government of India) - CTRI number: CTRI/2015/01/005419. Informed consent was obtained from all patients or their next of kin before inclusion into the study.

**Fig. 1:**
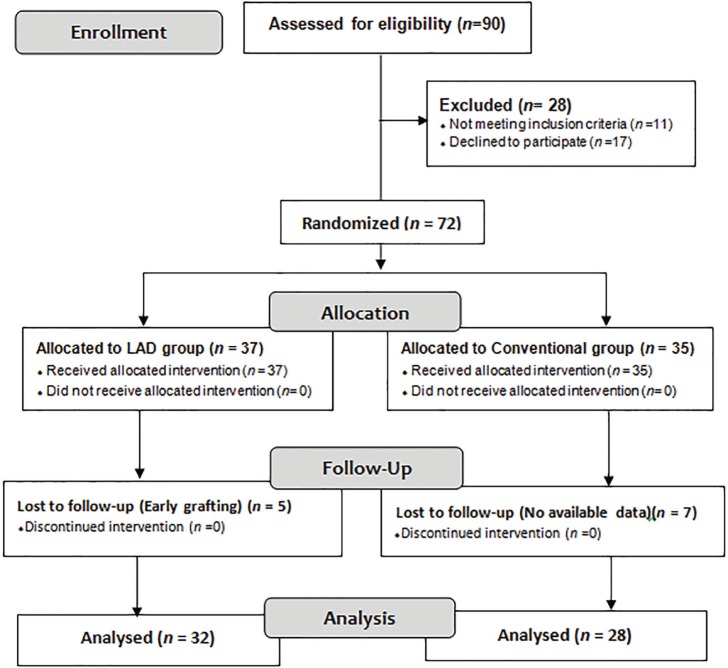
consort flow chart.

Ninety patients of age 12 to 65 years (Mean age: 38.5) ailing from burn injury were enrolled into the study. After examined inclusion criteria [10-30% total body surface area (TBSA) burn wounds] and exclusion criteria (patients with collagen disorders, diabetic patients, leprosy patients, pregnant women, liver cirrhosis, HIV +ve status) and the cases had mean granulating wound of 18% (range10-30%) at the time of inclusion in the study. Seventy two patients were randomized of whom 37 and 35 were assigned to the LAD group (n=37), and conventional dressing group (n=35) by simple randomization (Figure 1) generating tables of random numbers through www.random.org. 

Numbers were assigned to a treatment group and sealed in opaque envelopes containing labelled paper with treatment and patient ID. On 0^th ^day, biopsies taken from both groups, LAD group- patients were treated with LAD with intermittent negative pressure. Conventional closed dressing group-patients were dressed daily with 5% povidone iodine solution soaked gauze. Wounds were washed daily both LAD and conventional group prior to dressing by povidone iodine solution. Out of 72 patients, twelve participants (Five in the LAD group and seven in the conventional dressing group), were withdrawn from the study before biopsies were taken. In Sixty patient biopsies were taken on 10^th ^day and were analyzed for biochemical histopathologic parameters under study. 

Standard L-hydroxyproline, bovine serum albumin (BSA), standard glutathione, l-chloro-2,4-dinitrobenzene, nictoinamide adenine dinucleotide phosphate (reduced form), glutathione reductase (type III, Baker’s yeast), cumene hydrogen peroxide (Sigma–Aldrich, St. Louis, MO, USA), thiobarbituric acid, tri-chloroacetic acid, 1,1,3,3-tetramethoxypropane, all inorganic salts and organic reagents, graded alcohol, hematoxylin-eosin (Sigma-Aldrich, MO, USA).

The granulation tissue samples obtained from these subjects were used for the analysis. The wet weight of the tissues was noted and the tissues were dried at 60°C for 24 hours to record the constant dry weight. The dried tissues were treated with 10 mL 6N HCl and kept at 110°C for 24h. The neutralized acid hydrolysate of the dry tissues was used for determination of the hydroxyproline content by the method of Neuman and Logan. Granulation tissue samples wet weight was noted and homogenized by Rotex homogenizer in ice-cold 0.2 M phosphate buffer (pH: 7.4). Homogenates were centrifuged at 15,000 rpm for 30 min in cooling centrifuge and supernatant was then used for determine total protein, lipid peroxidation and GSH, GPX activity was determined. Protein concentration was determined according to Lowry et al. (1951) using purified bovine serum albumin as standard.

Tissue hydrolysate was prepared and used for estimation of hydroxyproline,^24^ total protein,^25^ lipid peroxidation,^26^ reduced glutathione,^27^ and glutathione peroxidase activity.^28^ Tissue preparation for histopathologic study wound biopsies performed on days 0 and 10 were collected, fixed in 10% formalin, dehydrated through an increasing alcohol series (50%, 70%, 90%, and 100%), cleared in xylene and embedded (Leica, EG1150 H) in paraffin wax (melting point: 56°C). Serial sections of 5 µm thickness were cut using a microtome (Leica, RM2255) and were stained with hematoxylin-eosin (Sigma-Aldrich, MO, USA). Each slide was given a histopathological score ranging from 1 to 12, with 1 corresponding to no healing and 12 corresponding to a completely reepithelialised wound.^29^

The scoring was based on the degree of cellular invasion, granulation tissue formation, vascularity, and reepithelialization. The histopathological score was assigned by investigator; code describing treatment to the patients was broken after the scoring was completed. Statistical analysis for biochemical parameters was performed by Student’s t-test and data were expressed as mean±standard deviation (SD). Histopathological score between the groups was performed by Student’s t-test and data were expressed as mean±standard error (SE) using the SPSS software (15^th^ version package, Chicago, IL, USA). A *p* value <0.05 was considered as significant. When appropriate, statistical uncertainty was expressed by the 95% confidence levels.

## RESULTS

Hydroxyproline level (75.2±26.30 µg/mg dry tissue weight), total protein level (15.6±8.23 mg/g wet tissue weight), GSH level (7.40±1.91 µg/mg protein), GPx (112.3±46.4 µmol/min/mg protein) were significantly higher in LAD group in comparison to the conventional group (27.8±15.5 µg/mg dry tissue weight, *p*=0.010), (10.26±4.94 mg/g wet tissue weight, *p*=0.003), (5.1±1.28 µg/mg protein, *p*=0.037) and (92±32.4 µmol/min/mg protein, *p*=0.016), respectively (Table 1). MDA level significantly decreased in LAD group (12.8±6.62 nmole/mg protein) when compared to conventional dressing group (6.6±3.7 nmole/mg protein, *p*=0.002). 

**Table 1:** Levels of hydroxyproline, total protein, MDA, GSH, GPX in granulation tissue of burn wound in LAD group & Conventional dressing group.

**Parameters**	**LAD Group (n=32) ** ** [Mean±SD]**			**Conventional dressing group (n=28)** ** [Mean±SD]**			***p*** ** value**
	**Day 0** ^th^	**Day 10** ^th^	**Day (0** ^th^ **-10** ^th^ **)**	**Day 0** ^th^	**Day 10** ^th^	**Day (0** ^th^ **-10** ^th^ **)**	
Hydroxyproline (µg/mg of dry weight of tissue)	61.7±11.7	136.9±24.2	75.2±26.30	69.8±9.7	97.6±17.2	27.8±15.5	0.010
Total protein (mg/g of wet weight of tissue)	10.2±2.9	25.8±7.2	15.6±8.23	11.74±2.6	22.0±5.0	10.26±4.94	0.003
MDA (nmole/mg protein)	19.3±6.66	6.5±2.24	12.8±6.62	17.2±5.6	10.6±3.8	6.6±3.7	0.002
GSH (µg/mg protein)	15.1±4.1	22.5±3.1	7.40±1.91	15.8±3.41	20.9±4.01	5.1±1.28	0.037
GPx ( **µMoles** NADPH oxidized/min/mg protein)	261.0±64.1	373.6±65.4	112.6±46.4	251.1±76.0	343.1±78.6	92±32.4	0.016

SD: Standard deviation; LAD: Limited access dressing; MDA: Malondialdhyde; GSH: Reduced glutathione; GPx: Glutathione peroxidase; NADPH: Nicotinamide adenine dinucleotide phosphate.

**Fig. 2:**
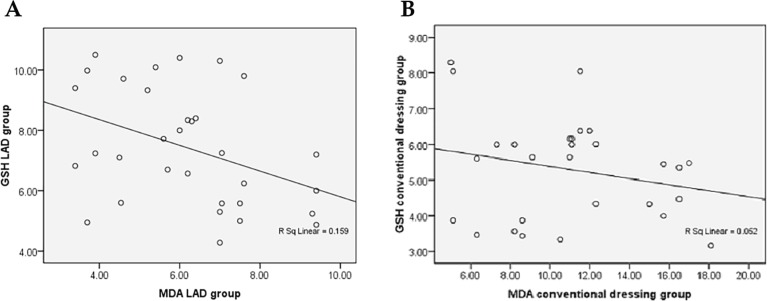
A: Correlation between GSH and MDA showing significant negative correlation in LAD Group (r=−0.399, *p*=0.024). B: Correlation between GSH and MDA showing no significant negative correlation in conventional dressing Group (r=−0.229, *p*=0.242).

In LAD group, a significant negative correlation was noted between GSH and MDA (Pearson correlation coefficient r=−0.399 *p*=0.024, Figure 2A), but in conventional dressing group, there was a negative correlation that was not significant (Pearson correlation coefficient r=−0.229, *p*=0.242) between GSH and MDA levels (Figure 2B). A stronger negative correlation between MDA (oxidative stress bio-marker: decreased) and GPX (anti-oxidant: increased) was noticed in LAD group when compared to conventional dressing group indicating a better protection of wound cells from ROS damage.

**Fig. 3:**
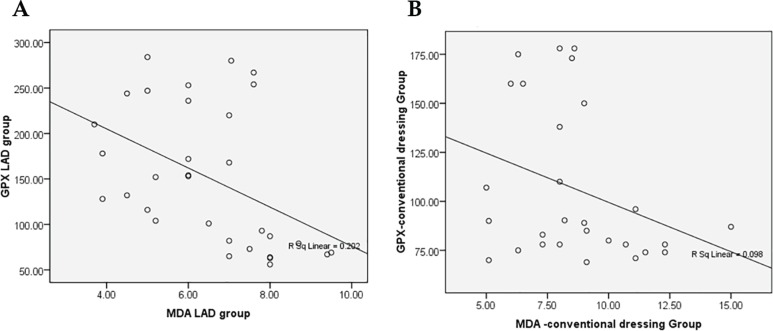
A: Correlation between GPX and MDA in LAD group showing significant negative correlation (r=−0.450, *p*=0.010). B: Correlation between GPX and MDA in conventional dressing group showing no significant negative correlation (r=−0.313, *p*=0.104).

In LAD group, a significant negative correlation between GPX with MDA (Pearson correlation coefficient r=−0.450, *p*=0.010, Figure 3A) was observed, but between GPX and MDA in conventional dressing group, there was a negative correlation that was not significant (Pearson correlation coefficient r=−0.313, *p*=0.104, Figure 3B). A stronger negative correlation between MDA (oxidative stress bio-marker: decrease) and GPX (anti-oxidant: increase) was seen in LAD group when compared to conventional dressing group indicating a better protection of wound cells from ROS damage.

**Fig. 4:**
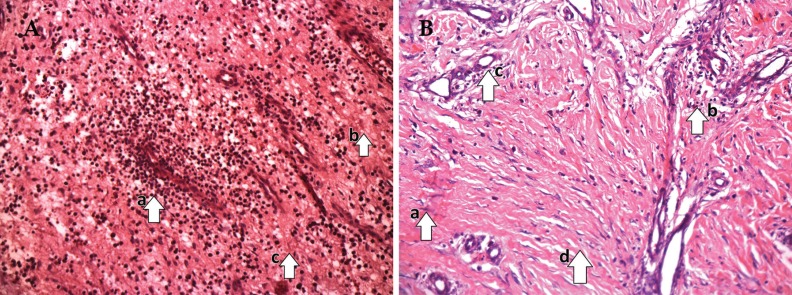
A: Pre-LAD (0^th^ day) appearance [(a) numerous neutrophils infiltration, (b) minimum number of fibroblasts, (c) fewer collagen fibers], B: Post-LAD (10th day) appearance [(a) maximum number of fibroblasts, (b) fewer inflammatory cells, (c) more proliferating blood capillaries (neovascularization), (d) Collagen bundles organized well between the cells] (Photograph with Olympus PM20 photomicroscope 20X magnification; H&E stain).

**Fig. 5:**
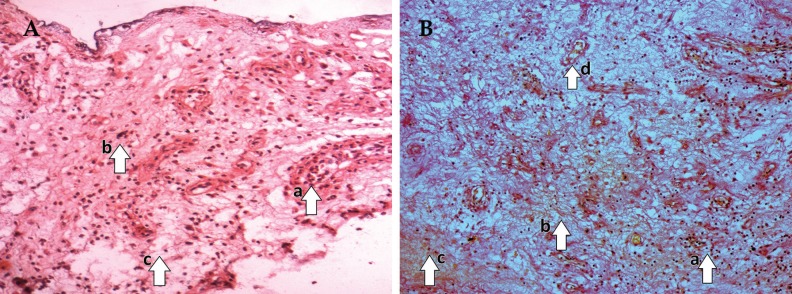
A: Pre-Conventional dressing (0^th^ day) appearance [ (a) numerous neutrophils infiltration, (b) minimum number of fibroblasts, (c) poor collagen fibers], B: Post-conventional dressing (10^th^ day) appearance [(a) numerous neutrophils infiltration, (b) poorly developed matrix minimum number of fibroblasts, (c) Poor collagen bundles] (Photograph with Olympus PM20 photomicroscope 20× magnification; H&E stain).

On 0^th^ day of treatment, LAD and conventional groups showed a necrotic tissue with increased cellular infiltration (Figure 4A and 5A). On 10^th^ day, LAD group showed an increase in ECM deposition, decrease in cellular infiltration and increased angiogenesis (Figure 4B) in comparison to that of the conventional dressing group (Figure 5B). Histopathological scoring on 0^th^ day and 10^th^ day, biopsies of granulation tissue of both groups were analyzed (Table 2). The average histological score of wounds was similar on 0^th^ day both in LAD vs. conventional dressing group (5.2±0.43 vs. 4.56±0.45, Figure 4A and 5A). 

**Table 2:** Histopathological grading score.

**Group **	**N**	**0** ^th^ ** day ** **(Mean ** **±** **SE** **)**	**10** ^th^ ** day** **(Mean ** **±** **SE** **)**	**(0-10** ^th^ **) day** **(Mean ** **±** **SE** **)**	***p *** **value**
LAD	32	5.2±0.43	8.70±0.21	3.5±0.36	0.015
Conventional dressing	28	4.56±0.45	6.24±0.40	2.3±0.29	

A Student’s t-test was performed to determine the significant difference in the average (Mean) histological score of LAD and conventional group. The result indicates that there was a significant difference in the average score of the two groups (*p*=0.015), LAD: Limited access dressing.

Wound healing was markedly improved in LAD group (8.70±0.21) after 10 days of treatment in comparison to conventional dressing group (6.24±0.40). The mean±SE on 0^th^–10^th^ day (LAD vs. conventional dressing group=3.5±0.36 vs. 2.3±0.29; *p*=0.015). Consistent with these findings, the histological score of granulation tissue of LAD group was significantly higher (Figure 4B) with decreased neutrophil filtration and increased fibroblasts, collagen deposition, increased the number of capillary vessels per high power field than conventional dressing group (Figure 5B).

## DISCUSSION

Thermal injury of the skin is an oxidation process, associated with biological and metabolic alterations; Free radical damage played a significant role in delaying wound healing and that ROS produced in response to cutaneous injury impeded the healing process by causing damage to cellular membranes, DNA, proteins, and lipids.^30^ Thermal injury generates free radicals from various cellular populations through many pathways; and the modulation of generated free radical activity with antioxidants seems to be an important part in treatment of burns.^31^

In burn patients alterations in the antioxidant micronutrient status and in the endogenous antioxidant defense against the deleterious effects of free radicals seem crucial, as pointed out by recent studies.^32^ A number of studies have proven that the advantages of NPWT in healing burn wounds,^33^ but there is lack of literature specifically stating about the reduction of the reactive oxygen species through NPWT and there is few literature available showing the effect of intermittent negative pressure dressing (LAD) on ROS and antioxidants.^34^

In the present study, the MDA, derived from the breakdown of fatty acid peroxide moieties, are be significantly decreased in to LAD group 12.8±6.62 nmole/mg protein compared to conventional dressing group 6.56±2.24 nmole/mg protein (Table 1). Higher level of MDA in conventional dressing group will have deleterious oxidative effects on membrane lipids^35^ result in poor granulation growth as observed clinically.^20^ Free radicals and their scavenging systems are known to play a important role in the normal and delayed healing of certain types of wounds.^36^ The magnitude of the generation of free radicals and their disposal mechanisms are known to be altered in burn patients, and some kind of correlation exists between altered free radical cascades and delayed wound healing.^7^

Enzymatic antioxidants such as GPx, GST, catalase levels play crucial role during burn wound healing.^4^ Therefore, estimation of antioxidants like GSH,GPx and protein content in granulation tissues is also relevant because these antioxidants hasten the process of wound healing by destroying the free radicals.^37^ In the present study, the mean±SD of GSH- 7.40±1.91, GPx- 112.3±46.4, total protein- 15.6±8.23 content in LAD Group were significantly higher than the conventional dressing group (Table 1).

Collagen content of the granulation in wounds can be measured by monitoring the concentration of hydroxyproline which is a marker of collagen biosynthesis.^38^ Higher concentration of hydroxyproline reflects faster rate of wound healing which indicates increased cellular proliferation and thereby increased collagen synthesis.^39^ Lower concentration of hydroxyproline indicates poor wound healing.^40^ Various studies on human wound models shown that dressing technique like mosit wound dressing^41^ and continuous NPWT^42^ increased the level of hydroxyproline content in wound granulation tissue.

In the present study the hydroxyproline content in LAD group was 77.32±30.19 (µg/mg of dry weight of tissue) significantly higher than conventional dressing group (32.33±16.18, *p*=0.026). On correlation between GSH and MDA revealed a significant negative correlation (Pearson correlation coefficient r=−0.399, *p*=0.024) in LAD Group (Figure 2A), but in conventional dressing group, there was no significant negative correlation (Pearson correlation coefficient r=−0.229, *p*=0.242, Figure 2B). Correlation between GPx and MDA revealed a significant negative correlation between GPX with MDA (r=−0.362 *p*=0.028, Figure 3A), but conventional dressing group, there was no significant negative correlation (Pearson correlation coefficient r=−0.265, p=0.124) between GPX and MDA level (Figure 3B). The significant negative correlation between GSH/GPx with MDA indicates significant reduction in oxidative stress in LAD group.

Various histological studies have shown that continuous NPWT increase in rate of granulation tissue formation, angiogenesis, and decreased inflammation.^43^ In our study wound healing was markedly improved in LAD group (8.70±0.21) after 10 days of treatment than compare to conventional dressing group (6.24±0.40). The mean ±SE on 0^th^–10^th^ day (LAD vs. conventional dressing group=3.50±0.36 vs. 2.3±0.29; *p*=0.015).

In conclusion, the results obtained in this study showed a significant reduction of oxidative stress in LAD group in thermally injured patients (decreased MDA, increased GPx, GSH, negative correlation between GPx/GSH with MDA) and observation was supported by increased levels of hydroxyproline and total protein, amount of ECM deposition, degree of angiogenesis, decreased neutrophil infiltration and necrotic tissue in LAD group. Results of our biochemical and histopathological study supported the clinical observation indicating better clinical effect of LAD on granulation tissue.
